# Molecular Characterization of Noroviruses Causing Acute Gastroenteritis Outbreaks among US Military Recruits, 2013–2023

**DOI:** 10.3201/eid3014.240307

**Published:** 2024-11

**Authors:** Melissa S. Anderson, Chanel A. Mosby-Tourtellot, Regina Z. Cer, Daniel J. Crouch, Ryan S. Underwood, Cailin S. La Claire, Roger W. Pan, Evelyn W. Mahugu, Hunter J. Smith, Kimberly A. Bishop-Lilly, Mathew D. Esona, Francisco Mimica Porras, Michelle Hartman-Lane

**Affiliations:** Naval Health Research Center, Operational Infectious Diseases Directorate, San Diego, California, USA (M.S. Anderson, D.J. Crouch, R.S. Underwood, C.A. La Claire, R.W. Pan, M.D. Esona, F.M. Porras, M. Hartman-Lane); General Dynamics Information Technology, San Diego (M.S. Anderson, R.S. Underwood, C.A. La Claire, R.W. Pan); Defense Threat Reduction Agency, Fort Belvoir, Virginia, USA (C.A. Mosby-Tourtellot); Naval Medical Research Command, Biological Defense Research Directorate, Fort Detrick, Maryland, USA (C.A. Mosby-Tourtellot, R.Z. Cer, K.A. Bishop-Lilly); General Dynamics, Silver Spring, Maryland, USA (E.W. Mahugu); Global Emerging Infections Surveillance Branch, Armed Forces Health Surveillance Division, Silver Spring (E.W. Mahugu, H.J. Smith); Uniformed Services University, Bethesda, Maryland, USA (H.J. Smith).

**Keywords:** Norovirus, acute gastroenteritis, AGE, viruses, enteric infections, US military, recruit training, whole-genome sequencing, WGS, United States

## Abstract

Norovirus is the leading cause of acute gastroenteritis (AGE) worldwide. Norovirus outbreaks at military facilities can cause loss of training and working days and increased healthcare utilization, affecting force readiness. During 2013–2023, we enrolled 2,304 US military recruits from 4 basic training facilities to investigate AGE outbreaks among this population. Among enrollees, we detected norovirus in 433 (18.8%) AGE cases, and norovirus caused 49 AGE outbreaks during our longitudinal study. On average, each norovirus case-patient missed 1.2 training days due to illness, and 6.2% required infusion care. Whole-genome sequencing of selected samples from each outbreak produced full-length genomes (6,989–7,787 bp) for 39 samples. Norovirus GII.4 Sydney was the most (12/39, 30.8%) identified genotype over the study period. Phylogenetic and comparative genomic analyses revealed that several outbreak strains were responsible for causing >1 outbreak, even across different training sites. Our findings can inform infection control practices at military installations and overall norovirus vaccine development.

In the United States, norovirus causes 20 million acute gastroenteritis (AGE) cases, 110,000 hospital visits, and 900 deaths annually (*1*,*2*). Because norovirus is highly infectious and frequently causes outbreaks, the virus is of particular concern for populations in crowded environments, including military installations (*3*). Norovirus outbreaks can cause lost training and working days, increased healthcare utilization, and disruptions to military missions and training exercises (*4*,*5*).

Norovirus is a nonenveloped, single-stranded, positive-sense RNA virus in the family *Caliciviridae* (*6*). Currently, 10 norovirus genogroups (GI–GX) and 49 genotypes are recognized (*7*). Genogroups I and II (GI and GII) are the major causes of norovirus infection in humans (*8*).

The Global Emerging Infections Surveillance Branch and Naval Health Research Center’s (NHRC) Operational Infectious Diseases (OID) Directorate have conducted AGE surveillance among US Department of Defense (DoD) recruit populations since 2011. We conducted a retrospective analysis to investigate the genomic composition of norovirus strains identified as the etiologic agents of AGE outbreaks at US military recruit training facilities during 2013–2023.

## Methods

### Study Design

Recruits meeting the case definition were enrolled in the AGE surveillance study during April 22, 2013–February 13, 2023. Participants signed consent forms at enrollment and their participation was voluntary. Upon enrollment, participants in AGE surveillance completed a case report form detailing demographics, clinical data, symptoms, and illness impact on training. The AGE case definition was >3 episodes of vomiting, diarrhea, or both within 24 hours; or >2 episodes of vomiting, diarrhea, or both within 24 hours along with >2 of the following symptoms: abdominal cramps, abdominal pain, fever, nausea, or blood or mucus in stool.

We conducted a retrospective study of AGE surveillance across 4 DoD recruit basic training facilities: Marine Corps centers in California and South Carolina, a Navy center in Illinois, and an Army center in Missouri. The AGE study protocol was approved for human research by the NHRC institutional review board (protocol no. NHRC.2011.0012) and includes norovirus genomic analysis. 

### Norovirus Case and Outbreak Definitions

We defined a norovirus case as a laboratory-confirmed sample of norovirus GI or GII by real-time reverse transcription PCR (rRT-PCR). We defined a sporadic case as a single norovirus case occurring >5 days after another case at the same location. We defined a cluster was 2 Norovirus cases occurring within 5 days of each other but >5 days after any other case at the same location. We defined an outbreak as >3 norovirus cases where each case occurred within 5 days of the previous case or after a case at the same location.

### Sample Collection and Storage

At the time of study enrollment, participants provided a stool sample or 2 rectal swab samples for clinical testing. Rectal swabs were stored in universal transport medium. Raw stool and rectal swabs were stored and shipped to NHRC-OID at 4°C.

### Nucleic Acid Extraction

We extracted total nucleic acid from stool or rectal swab suspensions by using the QIAamp Viral RNA Mini kit (QIAGEN, https://www.qiagen.com) according to the manufacturer’s protocol except for addition of 1 µL of MS2 bacteriophage to each sample before extraction as a control. We stored extracted nucleic acid from all samples at −80°C for future use.

### rRT-PCR and Whole-Genome Sequencing

We tested each sample by multiplexed rRT-PCR by using the Ag-Path One-Step RT-PCR Kit (Thermo Fisher Scientific, https://www.thermofisher.com) and primers specific for norovirus GI, GII, and MS2, following previously described conditions and procedures (*9*). We selected a single norovirus sample from each outbreak across the sites for whole-genome sequencing (WGS).

To ensure sufficient nucleic acid was available for sequencing, we prioritized selected norovirus samples with rRT-PCR cycle threshold values <26 and sufficient volume. We thawed samples stored at −80°C and processed for total nucleic acid extraction, as described above for WGS. We generated full-length norovirus GI and GII amplicons by following previously described conditions and procedures (*9*), with separate reactions for GI and GII primers (*10*). We used Nextera XT DNA Library Preparation Kit (Illumina, https://www.illumina.com) to prepare libraries, following the manufacturer’s instructions, then sequenced on the MiSeq (Illumina) system using the MiSeq Reagent version 2 Sequencing Kit (Illumina) for 151 paired-end reads.

### Norovirus WGS Data Analysis

We used the MetaDetector pipeline (*11*) to process raw reads. To remove adaptor and primer sequences and trim sequences based on quality scores, we used bbduk version 38.96 embedded in BBMerge software, then assembled the resulting data by using metaSPAdes and SPAdes version 3.15.3 (*12**–**14*). Then, we mapped reads back to contigs by using BBmap version 38.96 (*15*) and classified reads and contigs by using DIAMOND BLAST (*16*) against the National Center for Biotechnology Information (NCBI) nonredundant protein database (*15*,*17*). We used MEGAN (*18*) to analyze results from MetaDetector and note blastx (https://blast.ncbi.nlm.nih.gov) results for the largest assembled contig for each sample (*19*). We trimmed whole-genome nucleotide sequences to RNA-dependent RNA polymerase (RdRp) sequences and determined P-types by using blastn. To identify open reading frames (ORFs), we used the CLC Genomics Workbench (QIAGEN) to analyze nucleotide sequences representing the full-length genomes of 39 norovirus strains.

### Phylogenetic Tree Construction

We used the MUSCLE algorithm in MEGAX version 11.0 (*20*) to align consensus norovirus WGS and ORF2 sequences from the study strains, along with reference WGS nucleotide and protein sequences of norovirus GI and GII obtained from GenBank and CaliciNet (https://www.cdc.gov/norovirus/php/reporting/calicinet.html). We used MEGAX to construct phylograms of WGS and ORF2 sequences and calculated sequence identities on the basis of distance matrices prepared by using the p-distance algorithm in MEGAX (*20*).

## Results

Across the 4 training centers, AGE surveillance enrolled 2,304 military recruits in the study (Table 1). Of those, 433 (18.8%) were positive for norovirus GI, GII, or both (Table 1). We identified norovirus GII in 290 (67.0%) cases and GI in 142 (32.8%) cases. Of the 433 confirmed norovirus cases, recruits missed an average of 1.2 training days per case; 27 (6.2%) case-patients received infusion care, and 4 (<1.0%) were hospitalized (Table 1). No deaths were reported.

**Table 1:** Characteristics of AGE enrollments, NoV cases, and training impacts in a study of molecular characterization of noroviruses causing acute gastroenteritis outbreaks among US military recruits at 4 basic training facilities, 2013–2023*

Characteristics	California	South Carolina	Illinois	Missouri	Total
No. AGE enrollments	567	435	734	568	2,304
Norovirus genotypes detected					
GI/GII†	132 (23.3)	131 (30.1)	78 (10.6)	92 (16.2)	433 (18.8)
GI‡	54 (40.9)	37 (28.2)	26 (33.3)	25 (27.2)	142 (32.8)
GII‡	78 (59.1)	93 (71.0)	52 (66.7)	67 (72.8)	290 (67.0)
GI and GII‡	0	1 (0.8)	0	0	1 (0.2)
No norovirus detected†	433 (76.4)	290 (66.7)	650 (88.6)	471 (82.9)	1,844 (80.0)
Failed PCR†§	2 (0.4)	14 (2.3)	6 (0.8)	5 (0.9)	27 (1.2)
Norovirus cases					
Complete case report form‡	128 (97.0)	124 (94.7)	74 (94.9)	92 (100)	418 (96.5)
Missed 1–2 training days¶	111 (86.7)	106 (85.5)	62 (83.8)	66 (71.7)	345 (82.5)
Received infusion care¶	5 (3.9)	13 (10.5)	9 (12.2)	0	27 (6.5)
Hospitalized¶	1 (0.8)	0	2 (2.7)	1 (1.1)	4 (1.0)

*Values are no. (%). Percentages may not add to 100% due to rounding and MS2 control. The 4 facilities were Marine Corps centers in California and South Carolina, a Navy center in Illinois, and an Army center in Missouri. AGE, acute gastroenteritis. †Percentages are of AGE cases. ‡Percentages are of norovirus GI/GII cases. §PCR reactions negative for all norovirus GI/GII targets. ¶Percentage of norovirus GI/GII cases with complete case report form.

During the study period, 47 sporadic norovirus cases, 23 clusters, and 49 outbreaks occurred across all 4 sites (Table 2). We detected norovirus GI in 14 (28.6%) and GII in 35 (71.4%) of the outbreaks. In total, 340 norovirus cases were associated with outbreaks, and outbreaks had an average of 6.9 (SD 4.8) confirmed cases.

**Table 2:** Outbreak descriptions in a study of molecular characterization of noroviruses causing acute gastroenteritis outbreaks among US military recruits at 4 basic training facilities, 2013–2023*

Outbreak description†	California	South Carolina	Illinois	Missouri	Total
Sporadic	17	5	13	12	47
Cluster	5	6	4	8	23
Outbreak	13	17	6	13	49
Norovirus GI outbreak, no. (%)	5 (38.5)	5 (29.4)	1 (16.7)	3 (23.1)	14 (28.6)
Norovirus GII outbreak, no. (%)	8 (61.5)	12 (70.6)	5 (83.3)	10 (76.9)	35 (71.4)
No. confirmed NoV cases from all outbreaks	105	114	57	64	340
Mean cases per outbreak (SD)	8.1 (5.8)	6.7 (4.1)	9.5 (7.0)	4.9 (2.5)	6.9 (4.8)
Combined length of outbreaks, d	90	98	56	90	334
Mean length per outbreak, d (SD)	6.9 (5.0)	5.8 (+4.9)	9.3 (8.3)	6.9 (5.5)	6.8 (5.5)

*The 4 facilities were Marine Corps centers in California and South Carolina, a Navy center in Illinois, and an Army center in Missouri. †Sporadic = 1 norovirus case occurring >5 days after another norovirus case at the same location; cluster = 2 norovirus cases occurring within 5 days of each other but >5 days after any other norovirus case at the same location; and outbreak >3 cases occurring >5 days after another norovirus case at the same location.

### WGS Data Analysis

We selected a single Norovirus-positive sample from each outbreak for retrospective genomic analyses. Of the 49 selected samples, we were able to assemble 39 WGS sequences with lengths of 6,989–7,787 bp (Appendix 1 Table). Ten samples failed to produce quality norovirus genomes, and we removed those from further analyses. Norovirus GII.4 was the most identified genotype, causing 12 outbreaks across 3 sites (Appendix 1 Table). Overall, 12/39 (30.8%) sequences were identified as GII.4 Sydney. Norovirus GI.3 was the most identified GI genotype, causing 4 outbreaks across 2 sites (Appendix 1 Table).

### Phylogenetic and Sequence Analyses

**Figure 1:**
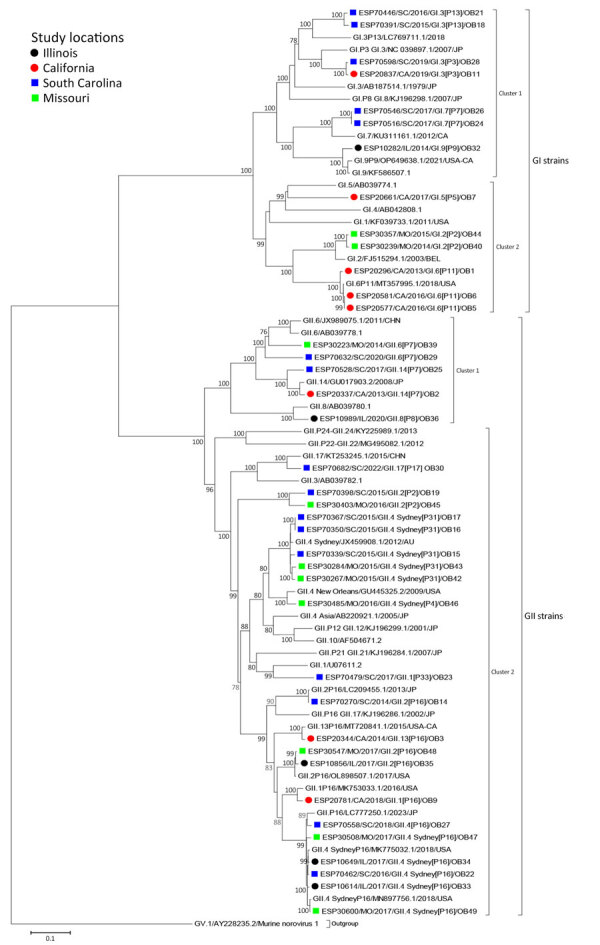
Maximum-likelihood phylogram in a study of molecular characterization of noroviruses causing acute gastroenteritis outbreaks among US military recruits at 4 basic training facilities, 2013–2023. Phylogram reveals genetic relatedness of whole-genome sequences (+7,500 bp) for 39 of the 49 selected outbreak virus study strains characterized by whole-genome analysis. Outbreak study strains are labeled from left to right as follows: sample identification/location sample was collected/year sample was collected/genogroup and P-type/outbreak number. The 4 facilities were Marine Corps centers in California and South Carolina, a Navy center in Illinois, and an Army center in Missouri. Bootstrap values >70% are indicated at branch nodes where applicable. Scale bar indicates nucleotide substitutions per site.

**Figure 2:**
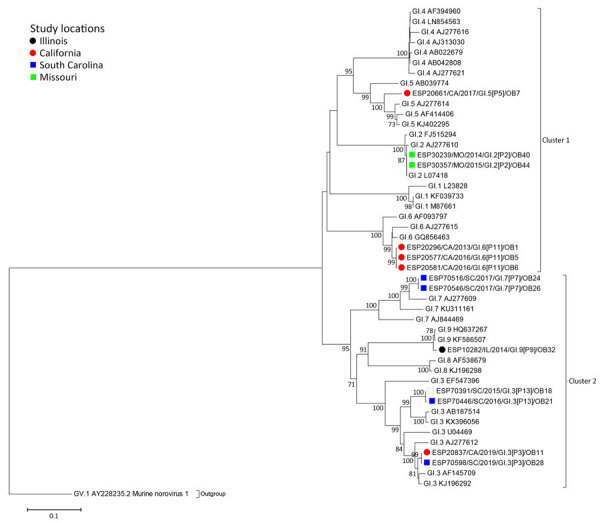
Maximum-likelihood phylogram of GI ORF2 (VP1) in a study of molecular characterization of noroviruses causing acute gastroenteritis outbreaks among US military recruits at 4 basic training facilities, 2013–2023. Phylogram reveals genetic relatedness of GI ORF2 (VP1) deduced amino acid sequences (+550 bp) for 13 of the 39 norovirus outbreak strains. Outbreak study strains are labeled from left to right as follows: sample identification/location sample was collected/year sample was collected/genogroup and P-type/outbreak number. The 4 facilities, identified by color, were Marine Corps centers in California and South Carolina, a Navy center in Illinois, and an Army center in Missouri. Bootstrap values >70% are indicated at branch nodes where applicable. Scale bar indicates nucleotide substitutions per site. ORF, open reading frame; VP, virus capsid protein.

**Figure 3:**
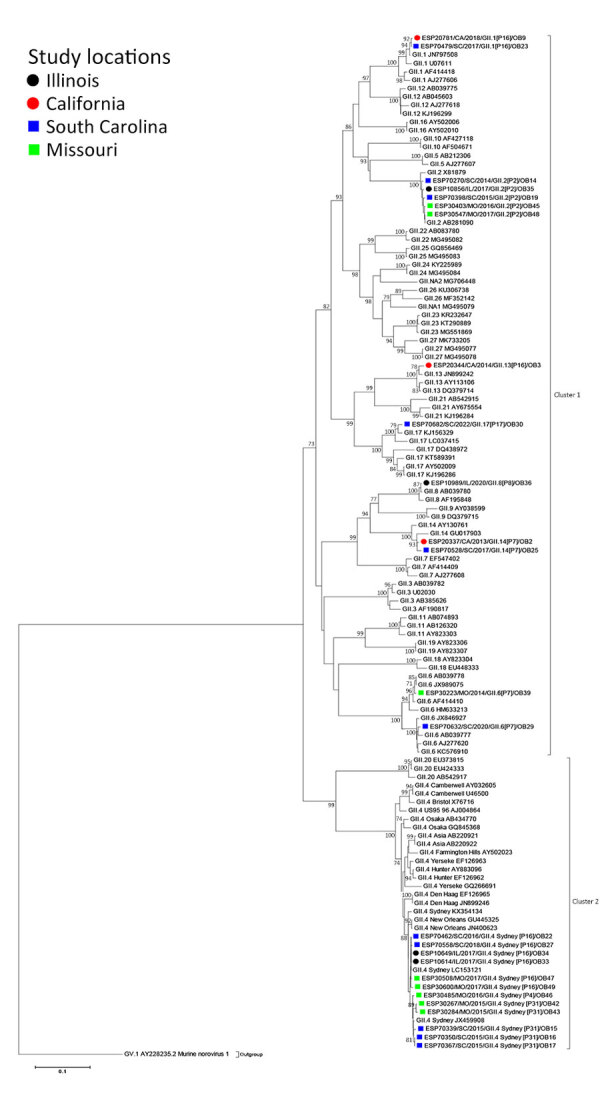
Maximum-likelihood phylogram of GII ORF2 (VP1) in a study of molecular characterization of noroviruses causing acute gastroenteritis outbreaks among US military recruits at 4 basic training facilities, 2013–2023. Phylogram reveals genetic relatedness GII ORF2 (VP1) deduced amino acid sequences (+580 bp) for 26 of the 39 norovirus outbreak strains. Outbreak study strains are labeled from left to right as follows: sample identification/location sample was collected/year sample was collected/genogroup and P-type/outbreak number. The 4 facilities, identified by color, were Marine Corps centers in California and South Carolina, a Navy center in Illinois, and an Army center in Missouri. Bootstrap values >70% are indicated at branch nodes where applicable. Scale bar indicates nucleotide substitutions per site. ORF, open reading frame; VP, virus capsid protein.

We used WGS data to perform phylogenetic and similarity matrix analysis of the 39 norovirus outbreak study strains and ORF2 sequences (Figures 1–3; Appendix 2). We further segregated the norovirus strains in both WGS GI and GII groups according to the genotypes and P-types. Within the GI group, we identified 2 paraphyletic clusters, and study and reference strains occupied the same cluster (Figure 1). Norovirus GI cluster 1 consisted of 7 study strains grouped according to genotypes with published GenBank strains. We observed the highest (99.9%) similarity between South Carolina strains ESP70516.V/SC/2017/GI.7[P7]/OB24 and ESP70546.V/SC/2017/GI.7[P7]/OB26 (Appendix 2 Table 1). Norovirus GI cluster 2 consisted of 6 study strains and previously reported strains retrieved from the GenBank database. Within cluster 2, three of the California study strains, ESP20296.V/CA/2013/GI.6[P11]/OB1, ESP20581.V/CA/2016/GI.6[P11]/OB6, and ESP20577.UTM/CA/2016/GI.6[P11]/OB5, were closely related phylogenetically and shared a high similarity (range 98.3%–100%) but were distantly related (72.1%–80.4%) to GenBank strains included in the analysis.

Norovirus GII strains segregated into 2 major clusters (Figure 1). Cluster 1 consisted of 5 study strains grouped according to genotypes and 3 GII reference strains. Within cluster 1, the study strains were distantly related (Appendix 2 Table 2). Norovirus GII cluster 2 included 21 study strains grouped according to genotypes along with several reference strains. Several study strains from South Carolina and Missouri clustered phylogenetically and shared high similarity with each other. We also noted high similarity (99%) between Illinois study strains ESP10614.V/IL/2017/GII.4 Sydney[P16]/OB33 and ESP10649.UTM/IL/2017/GII.4 Sydney[P16]/OB34.

The norovirus GI ORF2 sequences were phylogenetically segregated into 2 major clusters and were separated according to genotypes (Figure 2). GI ORF2 cluster 1 consisted of 6 study strains, and cluster 2 included 7 study strains grouped together with GenBank strains (Figure 2; Appendix 2 Table 3). Study strains ESP20296.V/CA/2013/GI.6[P11]/OB1, ESP20577.UTM/CA/2016/GI.6[P11]/OB5, and ESP20581.V/CA/2016/GI.6[P11]/OB6 in cluster 1 were identical to each other. In cluster 2, study strains ESP20837.V/CA/2019/GI.3[P3]/OB11 and ESP70598.V/SC/2019/GI.3[P3]/OB28, as well as ESP70516.V/SC/2017/GI.7[P7]/OB24 and ESP70546.V/SC/2017/GI.7[P7]/OB26 shared an absolute amino acid similarity with each other.

Phylogenetically, norovirus ORF2 GII study strains and cognate gene sequences of reference strains used in this analysis mapped into 2 major clusters. GII strains were grouped according to their genotypes and P-types. GII ORF2 strains were segregated into cluster 1 and cluster 2. Cluster 1 consisted of 14 study strains spread across different genotype groups. Of note, several study strains demonstrated high amino acid similarity across geographic sites; strains from Missouri and South Carolina showed similarity, as did strains from Illinois and South Carolina. All GII.4 study strains shared a high (93.4%–100%) similarity with GII.4 reference strains (Appendix 2 Table 4).

## Discussion

This 10-year retrospective study characterized norovirus-related AGE outbreaks across DoD recruit training facilities. Norovirus GII.4 Sydney was the most (12/39, 30.8%) identified genotype observed in this study, which is consistent with outbreak data from CaliciNet covering 2013–2016 (Appendix 1 Table) (*21*). Further, a novel [P16] polymerase type emerged in November 2015 that was associated with GII.4, causing 60% of outbreaks during 2015–2016 (*21*). We identified GII.4 Sydney[P16] in this study in December 2016, and it was responsible for 6/12 (50%) outbreaks through January 2018 (Appendix 1 Table).

Phylogenetic analysis of the ORF2 sequences showed that the GI and GII study strains were segregated into small clusters according to genotypes. Those same norovirus strain segregation patterns have been reported in previous studies (*22*,*23*). Similarity distance analysis of the WGS and ORF2 sequences showed that study strains ESP70516.V/SC/2017/GI.7[P7]/OB24 and ESP70546.V/SC/2017/GI.7[P7]/OB26 from South Carolina were genetically similar and shared an absolute percentage identity, indicating that the same strain was responsible for causing those 2 outbreaks. Similarly, study strain ESP20837.V/CA/2019/GI.3[P3]/OB11 from California shared 99.2% similarity with strain ESP70598.V/SC/2019/GI.3[P3]/OB28 from South Carolina, suggesting that the same strain was responsible for both outbreaks in 2019. Those findings could indicate cross-country transmission between the 2 Marine Corps training centers.

Molecular epidemiologic studies suggest that norovirus GII.4 caused multiple AGE pandemics in persons of all ages (*24*,*25*). In the current study, we identified norovirus GII.4 genotypes as the cause of multiple outbreaks in South Carolina, Missouri, and Illinois. Phylogenetically, the GII.4 Sydney[P16] from those 3 sites and the GII.4 Sydney[P31] from South Carolina and Missouri clustered together (Figure 3), and GII.4 Sydney strains were detected across the globe in previous years (*24*,*26*–*28*). Those data suggest that the origins of the GII.4 Sydney[P16] and GII.4 Sydney[P31] strains detected in this study might have been imported internationally or were circulating locally before causing outbreaks at the military facilities in our study. Similarly, 5 GII.2[P2] strains from South Carolina, Missouri, and Illinois clustered together with reference strains from abroad (Figure 3) (*29*,*30*).

One limitation of this study was the small number of samples. On average, 5 samples were collected per outbreak, but using more samples for sequencing would have provided more detailed information regarding the genomic epidemiology of each outbreak. In addition, we used cycle threshold values <26 as the selection criteria, thereby reducing the number of samples eligible for sequencing. Another limitation was the lack of detailed questionnaire data and limited questionnaire administration. Including more detailed data points in the questionnaire, such as decreased work and training performance metrics, would have clarified the detrimental impacts of norovirus in this population. Similarly, administering questionnaires to trainees experiencing symptoms but declining study enrollment would have enhanced our knowledge of the extent and effects of outbreaks.

## Conclusions

Despite its limitations, this study enhances our knowledge of the genetic code of norovirus strains among US military recruit populations. Norovirus can quickly cause large outbreaks among trainee and deployed military populations, potentially causing decreased mission readiness for entire units at a time for several days. As illustrated in this study, the average norovirus case-patient missed multiple training days due to infection, and 6.2% required infusion care. However, those numbers do not capture the full spectrum of norovirus impacts, including decreased workdays or training performance, which likely are greater than reported. Maintaining surveillance systems and sample repositories with associated WGS information can be critical to developing effective preventive measures against norovirus, such as vaccines (*31*–*33*). 

In conclusion, understanding Norovirus epidemiology could help inform military public health practices and support military health readiness. Having near-real-time genomic information can assist infection control and preventive medicine teams in pinpointing the outbreak etiologies and transmission dynamics to mitigate active outbreaks and prevent future norovirus outbreaks. Our findings will enable us to characterize and monitor the spread of norovirus strains, anticipate future patterns, pinpoint outbreak sources, and advance vaccine technology to enhance public health response efficacy.

Appendix 1 Additional information on molecular characterization of noroviruses causing acute gastroenteritis outbreaks among US military recruits, 2013–2023.

Appendix 2 Genomic information in a study of molecular characterization of noroviruses causing acute gastroenteritis outbreaks among US military recruits, 2013–2023.
