# Supplementary material for: Molecular Characterization of Noroviruses Causing Acute Gastroenteritis Outbreaks among US Military Recruits, 2013–2023
Source: Emerg Infect Dis. 2024 Nov;30(Suppl 2):S71–9. doi: 10.3201/eid3014.240307 (PMC11559571; doi:10.3201/eid3014.240307)
Supplement: Appendix 1 — Additional information on molecular characterization of noroviruses causing acute gastroenteritis outbreaks among US military recruits, 2013–2023. [file 24-0307-Techapp-s1.pdf]

Article DOI: https://doi.org/10.3201/eid3014.240307

EID cannot ensure accessibility for supplementary materials supplied by authors. Readers who have difficulty accessing supplementary content should contact the authors for assistance.

Molecular Characterization of Noroviruses Causing Acute Gastroenteritis Outbreaks among US Military Recruits, 2013–2023

Appendix 1

Appendix 1 Table. Summary of WGS results for NoV-positive stool samples from outbreaks

Outbreak no.	Sample collection date	Sample ID	Site	Mapped NoV reads	WGS sequence length, bp	Average coverage	Phylogenetic tree name	WGS phylogenetic tree	ORF2 phylogenetic tree	RdRp phylogenetic tree	Updated nomenclature
OB32	2/15/2014	ESP10282	Illinois (IL)	602328	7746	9571.71	ESP10282.V/IL/2014/GI.9[P9]/OB32	GI	GI.9	GI.P9	GI.9[P9]
OB33	1/3/2017	ESP10614	Illinois (IL)	342200	7570	5631.36	ESP10614.V/IL/2017/GII.4 Sydney[P16]/OB33	GII	GII.4 Sydney	GII.P16	GII.4 Sydney[P16]
OB34	2/6/2017	ESP10649	Illinois (IL)	223567	7504	3713.31	ESP10649.UTM/IL/2017/GII.4 Sydney[P16]/OB34	GII	GII.4 Sydney	GII.P16	GII.4 Sydney[P16]
OB35	12/6/2017	ESP10856	Illinois (IL)	239797	7551	3878.16	ESP10856.V/IL/2017/GII.2[P16]/OB35	GII	GII.2	GII.P16	GII.2[P16]
OB36	1/15/2020	ESP10989	Illinois (IL)	580704	7563	9402.54	ESP10989.V/IL/2020/GII.8[P8]/OB36	GII	GII.8	GII.P8	GII.8[P8]
OB1	5/17/2013	ESP20296	California (CA)	267204	7617	4240.30	ESP20296.V/CA/2013/GI.6[P11]/OB1	GI	GI.6	GI.P11	GI.6[P11]
OB2	12/9/2013	ESP20337	California (CA)	257905	7528	4304.90	ESP20337.V/CA/2013/GII.14[P7]/OB2	GII	GII.14	GII.P7	GII.14[P7]
OB3	1/10/2014	ESP20344	California (CA)	579242	7532	9573.94	ESP20344.V/CA/2014/GII.13[P16]/OB3	GII	GII.13	GII.P16	GII.13[P16]
OB5	11/16/2016	ESP20577	California (CA)	419872	7666	6648.81	ESP20577.UTM/CA/2016/GI.6[P11]/OB5	GI	GI.6	GI.P11	GI.6[P11]
OB6	11/22/2016	ESP20581	California (CA)	308565	7694	4995.84	ESP20581.V/CA/2016/GI.6[P11]/OB6	GI	GI.6	GI.P11	GI.6[P11]
OB7	8/8/2017	ESP20661	California (CA)	82076	6989	1484.99	ESP20661.V/CA/2017/GI.5[P5]/OB7	GI	GI.5	GI.P5	GI.5[P5]
OB9	11/21/2018	ESP20781	California (CA)	107934	7122	1898.19	ESP20781.V/CA/2018/GII.1[P16]/OB9	GII	GII.1	GII.P16	GII.1[P16]

Outbreak no.	Sample collection date	Sample ID	Site	Mapped NoV reads	WGS sequence length, bp	Average coverage	Phylogenetic tree name	WGS phylogenetic tree	ORF2 phylogenetic tree	RdRp phylogenetic tree	Updated nomenclature
OB11	8/19/2019	ESP20837	California (CA)	121600	7751	1780.74	ESP20837.V/CA/2019/GI.3[P3]/OB11	GI	GI.3	GI.P3	GI.3[P3]
OB39	9/15/2014	ESP30223	Missouri (MO)	230595	7553	3789.26	ESP30223.V/MO/2014/GII.6[P7]/OB39	GII	GII.6	GII.P7	GII.6[P7]
OB40	12/12/2014	ESP30239	Missouri (MO)	154048	7622	2516.15	ESP30239.UTM/MO/2014/GI.2[P2]/OB40	GI	GI.2	GI.P2	GI.2[P2]
OB42	2/9/2015	ESP30267	Missouri (MO)	756307	7565	12436.34	ESP30267.V/MO/2015/GII.4 Sydney[P31]/OB42	GII	GII.4 Sydney	GII.P31	GII.4 Sydney[P31]
OB43	3/19/2015	ESP30284	Missouri (MO)	275545	7518	4359.14	ESP30284.V/MO/2015/GII.4 Sydney[P31]/OB43	GII	GII.4 Sydney	GII.P31	GII.4 Sydney[P31]
OB44	9/28/2015	ESP30357	Missouri (MO)	266182	7682	3954.28	ESP30357.V/MO/2015/GI.2[P2]/OB44	GI	GI.2	GI.P2	GI.2[P2]
OB45	1/6/2016	ESP30403	Missouri (MO)	416994	7559	6906.32	ESP30403.UTM/MO/2016/GII.2[P2]/OB4 5	GII	GII.2	GII.P2	GII.2[P2]
OB46	12/7/2016	ESP30485	Missouri (MO)	724525	7598	11749.90	ESP30485.V/MO/2016/GII.4 Sydney[P4]/OB46	GII	GII.4 Sydney	GII.P4	GII.4 Sydney[P4]
OB47	1/12/2017	ESP30508	Missouri (MO)	283039	7577	4553.17	ESP30508.V/MO/2017/GII.4 Sydney[P16]/OB47	GII	GII.4 Sydney	GII.P16	GII.4 Sydney[P16]
OB48	3/14/2017	ESP30547	Missouri (MO)	87804	7480	1229.17	ESP30547.V/MO/2017/GII.2[P16]/OB48	GII	GII.2	GII.P16	GII.2[P16]
OB49	12/4/2017	ESP30600	Missouri (MO)	460454	7559	7422.92	ESP30600.V/MO/2017/GII.4 Sydney[P16]/OB49	GII	GII.4 Sydney	GII.P16	GII.4 Sydney[P16]
OB14	1/28/2014	ESP70270	South Carolina (SC)	302106	7552	5007.09	ESP70270.V/SC/2014/GII.2[P16]/OB14	GII	GII.2	GII.P16	GII.2[P16]
OB15	2/23/2015	ESP70339	South Carolina (SC)	376391	7557	6236.44	ESP70339.V/SC/2015/GII.4 Sydney[P31]/OB15	GII	GII.4 Sydney	GII.P31	GII.4 Sydney[P31]
OB16	3/9/2015	ESP70350	South Carolina (SC)	166148	7601	2787.10	ESP70350.V/SC/2015/GII.4 Sydney[P31]/OB16	GII	GII.4 Sydney	GII.P31	GII.4 Sydney[P31]
OB17	3/25/2015	ESP70367	South Carolina (SC)	245433	7532	3963.91	ESP70367.V/SC/2015/GII.4 Sydney[P31]/OB17	GII	GII.4 Sydney	GII.P31	GII.4 Sydney[P31]
OB18	9/10/2015	ESP70391	South Carolina (SC)	211343	7781	3305.57	ESP70391.V/SC/2015/GI.3[P13]/OB18	GI	GI.3	GI.P13	GI.3[P13]
OB19	10/5/2015	ESP70398	South Carolina (SC)	1024960	7563	16284.28	ESP70398.V/SC/2015/GII.2[P2]/OB19	GII	GII.2	GII.P2	GII.2[P2]
OB21	8/16/2016	ESP70446	South Carolina (SC)	210710	7787	3319.75	ESP70446.V/SC/2016/GI.3[P13]/OB21	GI	GI.3	GI.P13	GI.3[P13]
OB22	12/13/2016	ESP70462	South Carolina (SC)	699985	7544	11258.54	ESP70462.V/SC/2016/GII.4 Sydney[P16]/OB22	GII	GII.4 Sydney	GII.P16	GII.4 Sydney[P16]

Outbreak no.	Sample collection date	Sample ID	Site	Mapped NoV reads	WGS sequence length, bp	Average coverage	Phylogenetic tree name	WGS phylogenetic tree	ORF2 phylogenetic tree	RdRp phylogenetic tree	Updated nomenclature
OB23	1/27/2017	ESP70479	South Carolina (SC)	253670	7514	4233.93	ESP70479.V/SC/2017/GII.1[P33]/OB23	GII	GII.1	GII.P33	GII.1[P33]
OB24	7/3/2017	ESP70516	South Carolina (SC)	410366	7779	6500.69	ESP70516.V/SC/2017/GI.7[P7]/OB24	GI	GI.7	GI.P7	GI.7[P7]
OB25	7/14/2017	ESP70528	South Carolina (SC)	221912	7526	3510.68	ESP70528.V/SC/2017/GII.14[P7]/OB25	GII	GII.14	GII.P7	GII.14[P7]
OB26	8/16/2017	ESP70546	South Carolina (SC)	209833	7682	3393.48	ESP70546.V/SC/2017/GI.7[P7]/OB26	GI	GI.7	GI.P7	GI.7[P7]
OB27	1/11/2018	ESP70558	South Carolina (SC)	590318	7562	9795.00	ESP70558.V/SC/2018/GII.4 Sydney[P16]/OB27	GII	GII.4 Sydney	GII.P16	GII.4 Sydney[P16]
OB28	3/6/2018	ESP70598	South Carolina (SC)	156531	7774	2514.98	ESP70598.V/SC/2019/GI.3[P3]/OB28	GI	GI.3	GI.P3	GI.3[P3]
OB29	2/3/2020	ESP70632V	South Carolina (SC)	1019715	7506	15612.76	ESP70632.V/SC/2020/GII.6[P7]/OB29	GII	GII.6	GII.P7	GII.6[P7]
OB30	4/5/2022	ESP70682	South Carolina (SC)	195897	7590	3157.84	ESP70682.V/SC/2022/GII.17[P17]/OB30	GII	GII.17	GII.P17	GII.17[P17]

*bp, base-pairs; ORF, open reading frame; RdRp, RNA-dependent RNA polymerase; WGS, whole-genome sequencing.
